# Prevalence, Spectrum, and Outcomes of Single Coronary Artery Detected on Coronary Computed Tomography Angiography (CCTA)

**DOI:** 10.1155/2019/2940148

**Published:** 2019-08-06

**Authors:** Rashid Al Umairi, Maryam Al-khouri

**Affiliations:** ^1^Department of Radiology, The Royal Hospital, Muscat, Oman; ^2^Oman Medical Specialty Board, Muscat, Oman

## Abstract

**Background:**

Single coronary artery (SCA) is a rare congenital anomaly in which there is an isolated coronary artery that arises from a single coronary ostium and provides coronary blood supply to the entire myocardium. SCA is classified into different types based on the origin, branching pattern, and course. Although the majority of patients with SCA are asymptomatic, some patients can present with life-threatening symptoms.

**Aim:**

To examine the prevalence, anatomical distribution, and outcome of the single coronary artery anomaly detected on coronary computed tomography angiography (CCTA) in a single center in Oman.

**Methods:**

Retrospectively, we reviewed 4,445 patients who underwent coronary computed tomography angiography between September 2012 and August 2018 at the National Heart Center, Muscat, Oman. We identified patients with a single coronary artery, and we evaluated the origin, course, and outcome of SCA.

**Results:**

We found 12 patients with single coronary artery among 4,445 patients with a mean age of 56.4 years (age range: 34 to 71 years; male : female ratio: 5 : 7). The most common class was RIII-C seen in 4 patients. Other SCA included RII-C, RII-A, and RII-S, two in each class. One patient had RI and one had LII-P. Two patients had coronary artery bypass graft. No major adverse cardiac events were reported over a mean follow-up of 25.3 months.

**Conclusion:**

Single coronary artery (SCA) is a rare congenital anomaly classified into different types. In our study, the prevalence of SCA was 0.27% that is higher than the figures from previous reports.

## 1. Introduction

Single coronary artery (SCA) is a congenital anomaly that is usually discovered incidentally and has an estimated incidence ranging between 0.024% and 0.066% among patients undergoing routine coronary artery catheterization [1]. SCA can be either an isolated anomaly or associated with other congenital abnormalities such as coronary artery fistula and bicuspid aortic valve [2–4]. Based on the site of origin and anatomical distribution of the branches, Lipton et al. classified SCA into two main categories: “R,” right type, and “L,” left type. These two types were further divided into I, II, and III subtypes according to the anatomical courses of the branches [5]. Although conventional coronary angiography is the gold standard procedure for coronary artery assessment, it is an invasive procedure and has limited ability in demonstrating the anatomy of complex coronary artery anomalies. In contrast, CCTA is a noninvasive test and has the ability to show complex coronary artery anatomy [6]. The aim of this study is to discuss the prevalence, anatomical distribution, and outcome of singe coronary artery anomaly detected on coronary computed tomography angiography (CCTA) in a single center in Oman.

## 2. Patients and Methods

The Scientific Research Committee of The Royal Hospital, Muscat, Oman, approved this retrospective single institution study and waived informed consent.

### 2.1. Patient Selection

This study included 4,445 consecutive patients who underwent CCTA at the National Heart Center, Muscat, Oman, from September 2012 to August 2018. The indications for performing CCTA were chest pain to exclude coronary artery disease, assessment of coronary artery grafts or stents, cardiomyopathy, evaluation of congenital heart disease, evaluation of syncope, and precardiac surgery assessment. Exclusion criteria for CCTA were a history of prior allergy to iodinated contrast material, impaired renal function (estimated glomerular filtration rate less than 45 mL/min/1.73 m^2^), inability to follow the breathing instruction, uncontrolled heart rate, cardiac arrhythmia, and severe coronary calcification in calcium score scan.

Prior to the scan, electrocardiogram (ECG), heart rate, and blood pressure of all patients were checked. Procedure preparation included administration of 25–100 mg of atenolol orally to all patients with a baseline heart rate >70 beats/min to lower the heart rate. Sublingual nitroglycerine (0.8 mg) was administered to all patients 1 minute prior to the injection of the contrast unless it was contraindicated. Scanning was performed using a dual-source 256-slice (2 × 128) CT scanner (SOMATOM Definition Flash, Siemens, Erlangen, Germany) with rotation time 280 ms and a dual-source 384-slice (2 × 192) CT scanner (SOMATOM Force, Siemens, Erlangen, Germany) with rotation time 250 ms. All scans started with topogram, followed by a prospective ECG-gated nonenhanced CT scan at 75% of RR interval. The CCTA scan was performed by injecting 60 to 75 mL of contrast followed by 30 mL of saline solution at a rate of 6 mL/s. In the contrast-enhanced scan, ECG-gated prospective or retrospective scan with a slice thickness of 0.6 mm was acquired during breath holding in inspiration. The scan parameters were adjusted automatically or manually to acquire the best quality with the least radiation dose.

Data from the scanners were sent to separate image-processing working stations (syngo.via, Siemens, Berlin, Germany), and coronary arteries were assessed for atherosclerotic disease, anomalous origin, course, and termination. The single coronary artery is defined as an isolated coronary artery arising from a single coronary ostium in the absence of a second coronary ostium. Patients with a single coronary artery were selected, and images were again analyzed. SCA was categorized into different categories based on modified Lipton's classification.

## 3. Results

**Figure 1:**
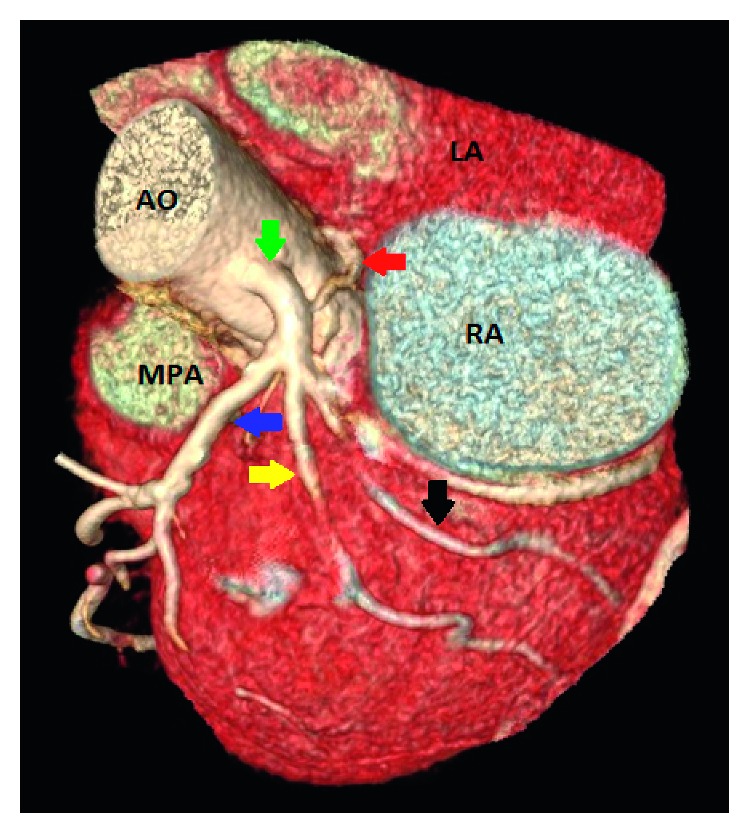
Case 3: 3D volume-rendered coronary image showing a single coronary artery arising from the left coronary sinus (LCS). The right coronary artery (RCA) (red arrow) arises from the proximal left main coronary artery, and then it courses posterior to the aorta (retroaortic course) before it reaches the right interventricular groove. The left main coronary artery trifurcates and gives the left anterior descending artery (blue arrow), the ramus intermedius (yellow arrow), and the left circumflex artery (black arrow). AO: aorta; LA: left atrium; RA: right atrium; MPA: main pulmonary artery.

**Figure 2:**
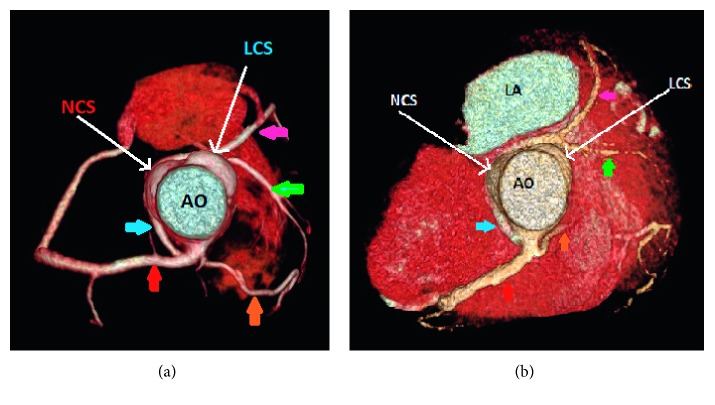
Case 1 (a) and case 9 (b): 3D volume-rendered coronary image showing a single coronary artery arising from the right coronary sinus (RCS) with a dual left anterior descending artery (LAD) variant. The right coronary artery (red arrow) has a normal course. The left main coronary artery (blue arrow) arises from the proximal RCA, and then it has a retroaortic course to the left before it gives the short segment of the LAD (green arrow) and the left circumflex artery (pink arrow). The long segment of the dual LAD variant arises from the proximal RCA, and it has a prepulmonic course in case 1 and transseptal course in case 9 before it reaches the distal anterior interventricular groove (orange arrow). AO: aorta; LCS: left coronary sinus; NCS: none coronary sinus; LA left atrium.

**Figure 3:**
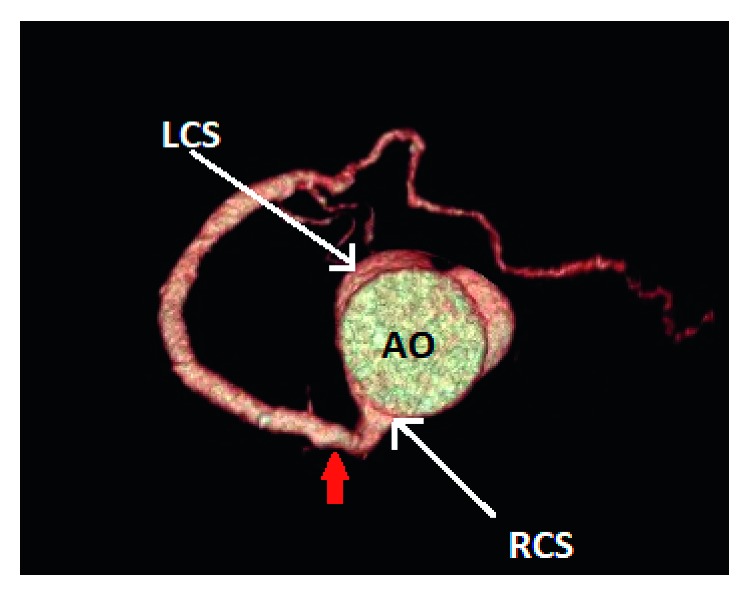
Case 2: 3D volume-rendered coronary image showing a single coronary artery (red arrow) arising from the right coronary sinus (RCS). The right coronary artery courses within the right atrioventricular groove before it continues within the left atrioventricular groove as the left circumflex artery. AO: aorta; LCS: left coronary sinus.

**Figure 4:**
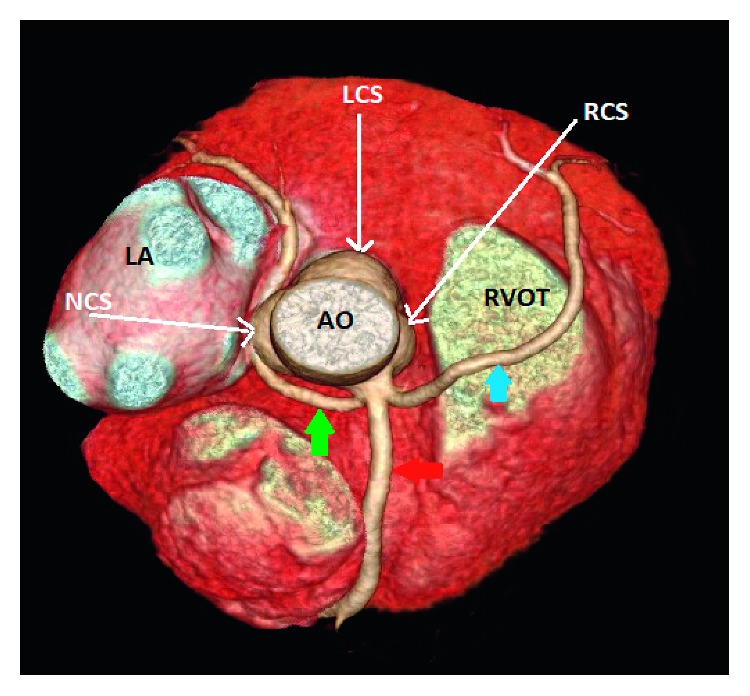
Case 3: 3D volume-rendered coronary image showing a single coronary artery arising from the right coronary sinus (RCS). The right coronary artery (RCA) (red arrow) has a normal course. The left anterior descending artery (blue arrow) arises from the proximal RCA and then courses to the left anterior to the right ventricular outflow tract (RVOT). The left circumflex artery arises from the proximal RCA, and then it has a retroaortic course before it reaches the left atrioventricular groove. AO: aorta; LCS: left coronary sinus; NCS: none coronary sinus; LA: left atrium.

**Figure 5:**
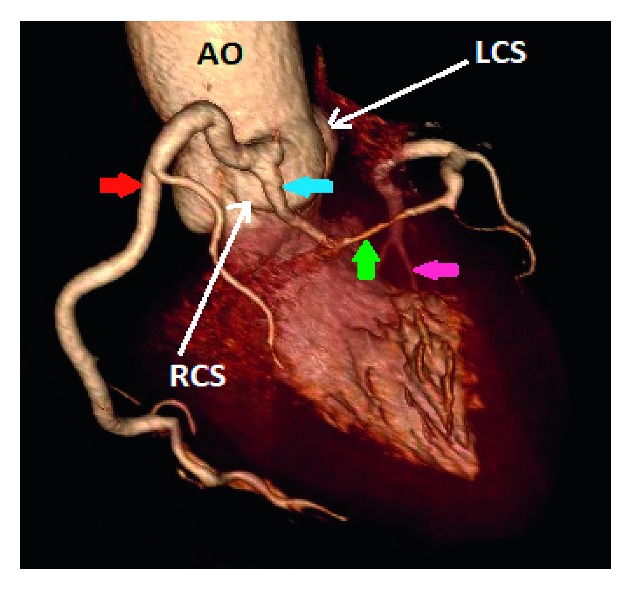
Case 4: 3D volume-rendered coronary image showing a single coronary artery arising from the right coronary sinus (RCS). The right coronary artery (red arrow) has a normal course. The left main coronary artery (blue arrow) arises from the proximal RCA and then courses to the left through the septal myocardium (transseptal) before it emerges in the proximal left interventricular groove and gives rise to the left anterior descending artery and left circumflex artery (pink arrow). There is significant narrowing of the transseptal segment of the left main coronary artery (green arrow). AO: aorta; LCS: left coronary sinus.

**Figure 6:**
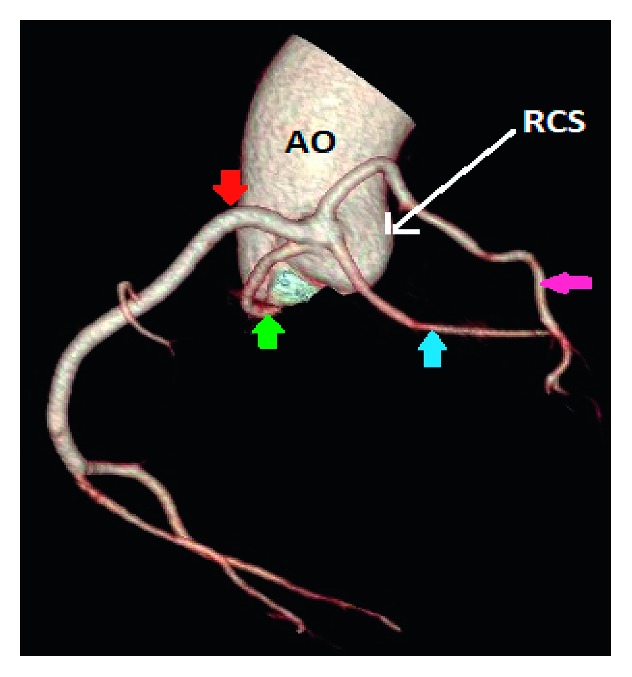
Case 6: 3D volume-rendered coronary image showing a single coronary artery arising from the right coronary sinus (RCS) with a dual left anterior descending artery (LAD) variant. The right coronary artery (red arrow) has a normal course. The left circumflex artery (green arrow) arises from the proximal RCA, and then it has a retroaortic course before it reaches the left interventricular groove. The short segment of the LAD courses through the septum myocardium before it reaches the proximal interventricular groove (blue arrow). The long segment of the LAD (pink arrow) has a prepulmonic course before it reaches the distal interventricular groove. AO: aorta.

We identified 12 patients with a single coronary artery with a prevalence of 0.27% (Table 1). Their mean age was 56.4 years (age range: 34 to 71 years; male : female ratio: 5 : 7). Of the 12 patients with SCA, only one patient had SCA originating from the left coronary sinus (Figure 1) and 11 had SCA originating from the right coronary sinus (Figures 2–6). Four patients had a dual LAD variant (Cases 1, 3, 6, and 9). None of the patient had any other congenital cardiac anomalies.

Chest pain was the most common symptom reported in ten patients. Eight patients had a history of shortness of breath on exertion, and five patients had a history of palpitation. The baseline clinical characteristics of the patients are shown in Table 1.

Four patients had myocardial perfusion imaging, two were negative for myocardial ischemia, one had an inconclusive test, and one had mild ischemia in the LAD territory (Case 8). One patient had an exercise treadmill test, and it was inconclusive (Case 11).

Coronary angiography was performed in nine patients which confirms the diagnosis of SCA. Three of them had no atherosclerosis (Cases 5, 6, and 11), two had mild (25–49%) coronary artery narrowing (Cases 4 and 8), and four had severe (≥70%) coronary artery stenosis (Cases 2, 3, 7, and 10). Two patients had coronary artery bypass graft because of severe coronary artery stenosis. The other patients with atherosclerotic disease were treated conservatively.

The mean time of follow-up was 23.3 months. None of the 12 patients had major adverse cardiovascular events during follow-up.

## 4. Discussion

Single coronary artery (SCA) is defined as an isolated coronary artery that arises from a single coronary ostium and provides blood supply to the entire myocardium [1]. SCA is usually detected incidentally while imaging the heart for other reasons, and it has a prevalence ranging between 0.024 and 0.098% among patients undergoing coronary computed tomography angiography [7–12]. The prevalence of SCA among our population was 0.27% that is higher than that previously reported. SCA can either be isolated or coexist with other cardiac congenital anomalies including transposition of great vessels, coronary arteriovenous fistula [2, 3, 13], tetralogy of Fallot, truncus arteriosus [14], interventricular septal defect, patent ductus arteriosus, bicuspid aortic valve, and patent foramen ovale [15–17]. None of our patients had other congenital cardiac anomalies. However, 4 of our patients had a dual LAD variant with the long and short LADs supplying the normal course of the LAD [18].

In the literatures, there are different classification systems of SCA based on necropsy findings and conventional coronary angiography. In 1979, Lipton et al. suggested a classification system in which they incorporated two prior classification systems that were suggested by Smith in 1950 and Ogden and Goodyer in 1970 [5, 19]. According to Lipton's classification, SCA is divided into two main types: “R,” right type, and “L,” left type, to indicate the origin of the SCA from the right or the left coronary sinus, respectively [5]. SCA is further divided into three subtypes based on the anatomical course of SCA. Type I in which there is a single coronary artery arising from either the right or the left coronary sinus is RCA, and then it continues in the left atrioventricular groove as LCX before it terminates as LAD, or as LM that gives LCX and LAD; the RCA formed is a continuation of the LCX. In type II, a single coronary artery arises from either the right or the left coronary sinus, and from the proximal segment of this single coronary artery, an anomalous artery arises that crosses the base of the heart before it resumes its inherited course. In type III, the LCX and LAD have separate origins from the proximal RCA [5]. Based on the relationship of the SCA to the aorta and the main pulmonary artery, Lipton et al. categorized SCA into three categories: category A in which the anomalous artery passes anterior to the main pulmonary artery, category B in which the anomalous artery passes between the ascending aorta and the main pulmonary artery, and category P in which the anomalous artery passes posterior to the ascending aorta [5]. In 1990, Yamanaka and Hobbs modified the classification proposed by Yamanaka and Hobbs and added two new groups S and C indicating transseptal and combined courses, respectively [20]. More recently, in 2005, Rigatelli et al. proposed a new classification system based on the clinical significance of the single coronary anomaly and categorized SCA into four different classes. Class I is considered a benign type, class II is associated with fixed myocardial ischemia, class III is related to sudden cardiac death, and class IV is associated with superimposed coronary artery disease [21]. In our survey, we had a variety of SCA subtypes with RIII-C as the most frequent type (Table 2).

Most of the patients with SCA are asymptomatic or might present with nonspecific symptoms; however, patients with some variants of SCA can present with typical chest pain, myocardial infarction, syncope, ventricular tachycardia, and sudden cardiac death [5, 15]. SCA with an interarterial anomalous artery has been strongly linked with sudden death and myocardial ischemia especially among young competitive athletes [15]. This is more common among patients with anomalous coronary artery arising from the right coronary sinus and coursing to the left between the aorta and the main pulmonary artery [22]. Although the exact mechanism is not fully understood, it is assumed that as the aorta and the main pulmonary artery become dilated during exercise, they squeeze the apparent artery [23]. Therefore, identification of patients with an SCA that has an interarterial course and is differentiated from the subpulmonic transseptal course is crucial for management.

SCA can be diagnosed by different diagnostic modalities including conventional coronary angiography, coronary computed tomography angiography, and cardiac MRI. Conventional coronary angiography is the gold standard for assessment of the coronary artery; however, it is an invasive procedure and has a risk of complications [6]. Moreover, even with multiple projections and angiographic views, delineation of the anatomy of the complex cases can be difficult [24]. On the contrary, coronary computed tomography angiography (CCTA) is a noninvasive diagnostic tool with high temporal and spatial resolution that has emerged as a gold standard for detection and characterization of coronary artery anomalies [6, 25, 26]. CCTA can precisely delineate the course of the anomalous artery and provide 3-dimensional information about the relation of the anomalous artery to other cardiovascular structures, namely, cardiac chambers and major arteries [13, 27]. However, despite the advances in CT technology to reduce radiation exposure, CCTA is still an ionizing examination. Cardiovascular MRI, on the contrary, is a nonionizing test that can be used to detect SCA and delineate the anatomy of the anomalous coronary artery along with the viability assessment of the myocardium; however, it has a lower temporal and spatial resolution compared to the CCTA. Moreover, MRI is less available than other diagnostic modalities and contraindicated in patients with pacemakers [28]. All our patients with SCA were diagnosed by CCTA, by which we were able to precisely delineate the origin and the course of the anomalous artery.

The presence of atherosclerotic disease in patients with coronary artery anomaly has a clinical implication, particularly when the decision is to be made between percutaneous coronary intervention (PCI) and coronary artery bypass graft (CABG). Moreover, awareness of presence of coronary artery anomalies and their anomalous course prior to CABG is crucial to avoid potential surgical complications as unrecognized anomalous artery can be excluded from perfusion during cardiopulmonary bypass or might be unintentionally damaged [15].

Given the vast variety of anatomical courses of SCA, the multidisciplinary approach involving interventional cardiologists and cardiothoracic surgeons should be considered to decide on the best treatment options. In the majority of asymptomatic patients with no atherosclerotic disease, usually, no invasive intervention is recommended [29]. Invasive interventions including PCI with stent placement and surgical intervention are preserved for symptomatic patients and patients with a malignant course of the anomalous artery [30, 31]. Surgical interventions include reimplantation of the anomalous artery to the aorta, osteoplasty, coronary artery bypass grafting (CABG) of the anomalous artery, and pulmonary artery translocation [32]. Two of our patients had signification coronary artery disease and underwent CABG.

## 5. Conclusion

The prevalence of SCA among our population is higher than that reported in other parts of the world. SCA is divided into different subtypes based on the course of the anomalous artery. Awareness of these types is essential as it has an implication for planning patient management. Coronary computed tomography angiography has an important role in diagnosis of different classes of SCA.

## Figures and Tables

**Table 1:** Baseline clinical characteristics of the patients with coronary artery anomalies.

Mean age (years)		56.4
Gender (M : F)		5 : 7
Risk factors	Hypertension	6
	Diabetes	5
	Dyslipidemia	5
	Smoking	3

Known CAD		2
Presenting symptoms	Chest pain	10
	Shortness of breath	8
	Palpitation	4

Intervention		2
Mean follow-up (months)		25.3

**Table 2:** 

	Classification	Invasive angiography	Myocardial perfusion	Exercise treadmill test	Stress echocardiogram
Case 1	RII-C	—	Inconclusive	—	−ve
Case 2	RI	Yes	−ve	—	−ve
Case 3	RII-C	Yes	—	—	Global hypokinesia
Case 4	RIII-C	—	—	—	Inferior wall hypokinesia
Case 5	RII-S	Yes	—	—	Lateral wall hypokinesia
Case 6	RIII-C	—	—	—	−ve
Case 7	RII-A	Yes	—	—	Wall motion abnormalities
Case 8	LII-P	—	Mild ischemia in LAD territory	—	—
Case 9	RIII-C	—	—	—	−ve
Case 10	RII-A	Yes	—	—	Hypokinesia
Case 11	RIII-C	Yes	−ve	Inconclusive	—
Case 12	RII-S	—	—	—	−ve

## Data Availability

The data used to support the findings of this study are included within the article.
